# Development of Telepresence Among Patients and Psychotherapists in the Actor-Partner Interdependence Model: Longitudinal Observational Study of 20 Dyads From a Randomized Trial on Video Consultations in Primary Care

**DOI:** 10.2196/70415

**Published:** 2025-08-11

**Authors:** Markus W Haun, Deborah van Eickels, Isabella Stephan, Justus Tönnies, Mechthild Hartmann, Michel Wensing, Joachim Szecsenyi, Andrea Icks, Hans-Christoph Friederich

**Affiliations:** 1 German Center for Mental Health (DZPG), Partner Site Mannheim-Heidelberg-Ulm, Heidelberg, Germany; 2 Department of General Internal Medicine and Psychosomatics, Heidelberg University, Im Neuenheimer Feld 410, Heidelberg, 69120, Germany, 49 622156 ext 8774; 3 Department of General Practice and Health Services Research, Heidelberg University, Heidelberg, Germany; 4 Heinrich-Heine-University, Centre for Health and Society, Institute for Health Services Research and Health Economics, Duesseldorf, Germany; 5 German Diabetes Centre, Institute for Health Services Research and Health Economics, Duesseldorf, Germany

**Keywords:** video consultations, telepresence, telehealth, integrated care, mental health, primary care, health services research

## Abstract

**Background:**

The COVID-19 pandemic has accelerated the adoption of video consultations in mental health care, highlighting the importance of therapeutic alliances for successful treatment outcomes in both face-to-face and web-based settings. Telepresence, the sense of being present with the mental health specialist (MHS) rather than feeling remote, is a critical component of building a strong therapeutic alliance in video consultations. While patients often report high telepresence levels, MHSs express concerns about whether video consultations can replicate the quality of face-to-face interactions. Despite its importance, research on telepresence development in MHSs over time and the dyadic interplay between patients and MHSs remains limited.

**Objective:**

This study aimed to evaluate the mutual influence within patient-MHS dyads on telepresence development during video consultations, using data from a randomized controlled trial assessing the feasibility of video consultations for depression and anxiety disorders in primary care.

**Methods:**

The study included 22 patient-MHS dyads (22 patients, 4 MHSs). Telepresence was measured using the Telepresence in Videoconference Scale. Dyadic data were analyzed using the actor-partner interdependence model with a distinguishable dyad structural equation model. Actor effects refer to the impact of an individual’s telepresence at time point 1 (T1) on their telepresence at time point 2 (T2), while partner effects represent the influence of one party’s telepresence at T1 on the other’s telepresence at T2. Sensitivity analyses excluded data from individual MHSs to account for their unique effects.

**Results:**

A significant actor effect for MHSs (*P*<.001) indicated a high temporal stability of telepresence between T1 and T2. In contrast, the actor effect for patients was not statistically significant, suggesting a greater variability between T1 and T2. No significant partner effects for both patients and MHSs were observed, suggesting no mutual influence between dyad members. Age was a significant covariate for telepresence in both groups.

**Conclusions:**

Consistent with prior findings, MHSs experienced increased telepresence over time, whereas patients reported high telepresence levels from the start of therapy. The lack of dyadic influence highlights the need for further exploration into factors affecting telepresence development, such as age, technical proficiency, and prior treatment experience. Future studies with larger samples and more sessions are necessary to enhance the generalizability of these findings and to optimize the use of video consultations in mental health care.

## Introduction

The COVID-19 pandemic has led to a significant increase in the use of video consultations in mental health care [1-4]. The therapeutic alliance, a critical factor for successful treatment outcomes in face-to-face therapy, is equally important in mental health specialist (MHS) video consultations [5-10]. Telepresence is defined as the sense of being present with the MHS in the same location rather than feeling remote [11-16]. Telepresence covers the following 3 domains: physical presence, social interaction, and absorption. Telepresence facilitates the working alliance when psychotherapy is delivered via videoconference. However, current evidence suggests its role is not essential. In fact, as noted by Bouchard et al [11], once a high level of telepresence is reached, it no longer predicts the strength of the working alliance. Patients frequently report high levels of telepresence [16-18], indicating that their experience during video consultations is comparable to face-to-face therapy [19,20]. In contrast, mental health professionals have expressed concerns about whether video consultations can achieve the same quality as traditional face-to-face therapies [21-23]. A comprehensive literature search (applying the search strings in Multimedia Appendix 1) up to June 1, 2025, revealed several studies on telepresence. However, most studies followed a cross-sectional design. Two surveys identified previous web-based consultation experience and perceived telepresence as predictors of mental health professionals’ comfort with video consultations [14,24]. One additional cross-sectional survey demonstrated that, besides previous experience with web-based consultations, the perceived level of telepresence was a significant predictor of mental health professionals’ level of comfort in the execution of web-based consultations [25]. Two experimental studies showed that stereo imaging, that is, enhancing the illusion of depth in an image, and emotionally charged discussions might boost telepresence [15,26]. We found only 2 clinical studies investigating telepresence. The findings of one study support the use of videoconferencing in the assessment of posttraumatic stress disorder [18]. Overall, we found only one study from 2007 that examined the development of telepresence over time [13]. This study linked telepresence to the patient-therapist bond across multiple sessions, treating patients with anxiety disorders. However, it did not measure telepresence in the participating MHSs. Currently, there is a lack of studies investigating the dyadic interaction between patients and therapists regarding the development of telepresence in both parties. While telepresence is traditionally conceptualized as a state-like, individually experienced phenomenon, emerging research suggests that it may be shaped within interpersonal exchanges, particularly in therapeutic contexts where presence, attunement, and alliance are coconstructed [27,28]. Breaks in alliance or disruptions in perceived presence are often mirrored or reciprocated, indicating that one party’s experience of telepresence may influence the other’s, even across sessions. Therefore, analyzing dyadic mutual influences over time is essential to understanding how stable or fluctuating patterns of telepresence unfold in therapeutic relationships.

This study aims to evaluate the mutual influence of the patient-MHS dyad on the development of telepresence during video consultations. We applied the actor-partner interdependence model (APIM) to dyadic data from a randomized controlled trial assessing the feasibility of MHS video consultations for depression and anxiety disorders in primary care [29]. By modeling both actor and partner effects across sessions, we aimed to evaluate whether an individual’s telepresence at one time point contributes to the development of the other’s telepresence at a subsequent time point—capturing potential delayed interpersonal effects in a therapeutic process that evolves over time.

## Methods

### Study Design and Setting

In a randomized pilot study, we enrolled 50 patients from 5 German primary care practices to assess the feasibility of an integrated mental health video consultation model for patients with depression and anxiety in primary care [30]. Primary care physicians were either recruited during a preceding qualitative preimplementation study or through a network of collaborating academic research practices affiliated with the Department of General Practice and Health Services Research at Heidelberg University [31,32]. We recruited 4 MHSs from the Institute for Psychotherapy in Heidelberg, a state-recognized training center affiliated with Heidelberg University. The specialists included clinical psychologists holding a diploma or master's degree enrolled in psychotherapy training, as well as physicians in residency programs working toward board certification in psychosomatic medicine and psychotherapy—an officially recognized medical specialty in Germany. All participants had completed a minimum of 2 years of professional training. The sample size of 50 patients was based on recommendations for obtaining reliable sample size estimates in feasibility studies [31,33]. Findings on feasibility, acceptability, and clinical effectiveness have been published elsewhere [31]. A total of 23 patients were allocated to the experimental group and received 4 video consultations. In contrast to studies cited in the introduction where telepresence was assessed following structured psychotherapy sessions, this study measured telepresence after more general clinical consultations between patients in the experimental group and MHSs. These consultations were part of an integrated care model that resembled traditional consultation-liaison and collaborative care approaches in mental health primary care. However, unlike purely consultative roles, the intervention incorporated more therapeutic components, such as the establishment of a working alliance, symptom monitoring, and, when appropriate, brief interpersonal-focused psychological interventions. As noted in prior work [15], the content and depth of a session may influence the perceived level of telepresence. While this study did not formally differentiate between consultation and therapy sessions, it is important to emphasize that the nature of the encounters in this trial represented a hybrid format, combining elements of both consultation and brief therapeutic engagement. This distinction is relevant when interpreting the levels and dynamics of telepresence observed. One patient allocated to the experimental group did not receive video consultations due to persistent connectivity failure. The remaining 22 patients from the intervention group and the 4 MHSs constitute the sample for the present analysis, resulting in a total number of patient-MHS dyads of 22. Due to attrition, the number of cases for patients on telepresence after the fourth video consultation was 20. Specifically, one patient dropped out expecting long-term therapy to be part of the intervention, and one patient dropped out for unknown reasons.

For dyadic data analysis, we used the APIM, which simultaneously estimates the effect of a person’s own variable (actor effect) and the effect of the same variable but from the partner (partner effect) on an outcome variable. The actor and partner variables are the same variable from different persons, and all individuals are treated as actors and partners. In this study, we investigated the effect of telepresence at time point 1 (T1) on telepresence at time point 2 (T2), that is, the end of the first and fourth video consultations, respectively. We treated the patient-MHS dyad members as distinguishable by the variable role [29,34]. We applied the Strengthening the Reporting of Observational Studies in Epidemiology (STROBE) Statement guidelines for reporting this study [35].

### Ethical Considerations

This study was conducted in accordance with the ethical standards of the Medical Faculty at the University of Heidelberg and with the 1964 Helsinki Declaration and its later amendments or comparable ethical standards. Ethics approval was obtained from the Ethics Committee of the Medical Faculty at the University of Heidelberg (Reference: S-634/2018). Informed consent was obtained from all individual participants included in the study. Participants were assured of the confidentiality of their responses and their right to withdraw at any time without penalty. We preregistered the study with the German Clinical Trials Register (DRKS00015812).

### Participants: Patients and Mental Health Specialists

Patients were recruited by their general practitioner. Inclusion criteria required patients to (1) exceed cut-offs of 9 points for the Patient Health Questionnaire-9, or for the Generalized Anxiety Disorder-7, respectively [36]; (2) currently have no or as yet insufficient treatment (psychotherapy, psychopharmacotherapy, or both) or difficulty with adherence; (3) agree to participate in the study by written informed consent; (4) be capable of giving consent; and (5) be 18 years or older. Patients were excluded if they (1) had substance misuse or dependence that was likely to compromise intervention adherence; (2) were acutely suicidal or put others at risk; (3) needed emergency medical treatment, such as hospital admission; (4) had acute psychotic symptoms, such as persecutory delusions or thought insertion; (5) had severe cognitive impairment or dementia; (6) had a substantial hearing or visual impairment; (7) were pregnant and in the second or third trimester; and (8) showed insufficient German language proficiency.

### Variables

We assessed the level of perceived telepresence by applying the 7-item Telepresence in Videoconference Scale (TVS) [37]. The TVS measures “the participants’ experience during the videoconference (eg, their interactions with the therapist, whether they felt as if they were physically present and actively participating during the session, etc)” [37]. The TVS consists of 3 factors (“Physical Presence” [items 1, 2, and 3], “Interaction” [items 4 and 5], and “Absorption” [items 6 and 7]), which explained 84.9% of the variance in the psychometric validation. The TVS comprises 7 items which are answered on a scale from 0 to 100 (“0%–completely disagree,” “50%–moderately agree,” and “100%–completely agree”) [37]. The total score is produced by calculating the mean of the 7 items. In the initial psychometric validation of the TVS, the estimate of internal consistency was within good limits (ie, Cronbach *α*=0.80; range in our total sample: 0.71 to 0.82), while the stability of the scale over time, that is, test**‐**retest reliability, was not measured in the original validation. However, a recent validation study with psychotherapists raised concerns regarding the “Absorption” subscale [38]. While “Physical presence” and “Interaction” showed good psychometric validity, the “Absorption” items substantially reduced the overall internal consistency, questioning the applicability of this subscale among professionals. In our sample, for MHSs, Cronbach α for the TVS was 0.77 at T1 and 0.81 at T2; Cronbach α for the absorption subscale was 0.60 at T1 and 0.71 at T2. Convergent and discriminant validity have been assessed through correlation with the Distance Communication Comfort Scale (*r*=0.42, *P*<.01) and the immersive tendencies questionnaire (*r*=0.0, *P*>.05), respectively. In this study, we applied the TVS total score as a mixed predictor variable at T1 and a mixed outcome variable at T2, respectively. We included age as a mixed continuous covariate varying both between and within dyads.

### Data Sources/Measurement

For patients, we collected demographic data and medical history using a structured self-administered questionnaire. For MHSs, we collected demographic data in brief interviews. We measured telepresence directly after each video consultation using computer-assisted telephone interviewing. The median interval between the initial and the final video consultation amounted to 49.5 days (IQR 35-59 days). We asked patients and MHSs to complete their questionnaires without discussing their answers with each other.

### Quantitative Variables

First, we implemented a dyadic structure of the data (ie, one row for every dyad, so-called wide format). Second, to facilitate the interpretation of the intercepts, we centered the predictor variables and covariates around their mean. We did not apply any groupings to the quantitative variables.

### Statistical Methods

For descriptive statistics, we summarized results for discrete variables in absolute and relative frequencies, while for continuous variables we provided means, SDs, medians, and IQRs. The data preparation for the multivariate analysis included screening for normality (univariate distributions). Specifically, we assumed multivariate normality if skewness and kurtosis item/score values fell in the normal range (−2 to 2 and −7 to 7, respectively). To investigate the missing data pattern, we ran Little’s MCAR test (R package BaylorEdPsych), which indicated a missing-completely-at-random pattern (*χ*^2^_2, N_*_=_*_176_=1.26; *P*=.53) and a maximum fraction of missing information of 2.3% at the item level. Hence, we applied full information maximum likelihood, which uses all available data and ensures valid inferences if the missingness is missing at random.

Next, to fit the APIM model to the data, we used the freely accessible web app APIM_SEM, which features structural equation modeling with maximum likelihood estimation based on the R package lavaan [34,39]. Given that the test for distinguishability was statistically significant, we used the model for distinguishable variables. The tests of coefficients were Z tests. Effect sizes for actor and partner effects were partial correlations. Betas were given twice, one using the overall SD across all persons for standardization and a second using the SD for patients and MHSs separately.

To account for the repeated-measures nature of the data (ie, each dyad contributes 2 time points per person, resulting in 4 observations per dyad), we used a version of the APIM tailored for longitudinal dyadic data. Specifically, the model was specified in a way that includes time as a repeated measure and models within-person stability (actor effects) as well as cross-partner influences (partner effects) over time. This approach treats repeated observations within dyads as nonindependent and accounts for the nesting of measurements within persons and dyads through the structural modeling of the residuals. Consequently, the statistical dependency resulting from repeated measurements and dyadic interdependence was addressed simultaneously, consistent with recommendations for longitudinal APIMs [29].

To control for measurement error in predictor variables and limit attenuation bias, we refitted the model correcting for such unreliability specifying the presumed reliability of the TVS at *α*=0.80. Before interpreting the APIM estimates, we checked model diagnostics (normality of residuals) and for potential outliers defined as cases with residuals of the fitted model that were more extreme than 4 SDs (absolute value). We did not identify any outliers. We performed a sensitivity analysis by excluding the data of one MHS at a time, to control for the effect of the individual MHSs. For all analyses outside APIM_SEM, we used R (version 4.5.0; R Core Team) and evaluated statistical significance at a type 1 error of 5% (2-tailed) [40].

## Results

### Main Results: Telepresence in Patient-Mental Health Specialist Dyads

#### Overview

**Figure 1.**
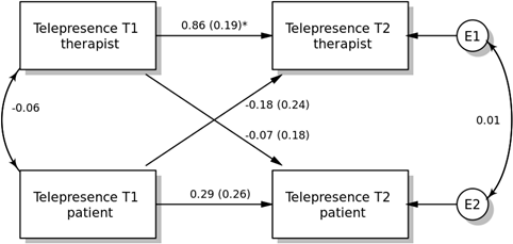
Actor-partner interdependence model with standardized parameter estimates. The double-headed arrow between “telepresence at T1 therapist” and “telepresence at T1 patient” represents its covariance. The double-headed arrow between “telepresence at T2 patient” and “telepresence at T2 therapist” is the residual nonindependence in these outcome scores, which is represented by the covariance between their corresponding 2 error terms. T1: time point 1; T2: time point 2. *P<.001.

The descriptive statistics of the raw variables are contained in Table 1, and the full information maximum likelihood estimated means and SDs in Table 2. The model converged after 125 iterations. Figure 1 contains the full APIM model. A summary of the results of the APIM analyses is provided in Table 3. The variance of the errors for the patients and MHSs was 138.89 and 125.94, respectively. The *R*² for the patients was 0.25, and for the MHSs, it was 0.58. The partial intraclass correlation for telepresence (TVS) at T2, controlling for the other predictor variables, was equal to .01 and was not statistically significant (*P*=.96, 95% CI −0.44 to 0.46). The difference in intercepts was equal to 3.88; this difference was not statistically significant (*P*=.48, 95% CI −6.92 to 14.68), which indicates that there was no main effect of role (patient/MHS).

**Table 1.** Descriptive summary of patients and mental health specialists.

	Patients in the intervention group (n=22)	Mental health specialists (n=4)
Age (years)		
Mean (SD)	45.1 (15.8)	38.3 (10.4)
Median (Min-Max)	48 (22.0-72.0)	35 (30.0-53.0)
Gender, n (%)		
Female	15 (68.2)	3 (75.0)
Male	7 (31.8)	1 (25.0)
Marital status, n (%)		
Single	4 (18.2)	1 (25.0)
In partnership	18 (81.8)	3 (75.0)
Education level, n (%)		
9 years or less	5 (22.7)	0 (0.0)
More than 9 years	15 (68.2)	4 (100.0)
Missing	2 (9.1)	0 (0.0)
Employment status, n (%)		
Employed	13 (59.1)	4 (100.0)
On sick leave	3 (13.6)	0 (0.0)
Retired	3 (13.6)	0 (0.0)
Unemployed	2 (9.1)	0 (0.0)
Missing	1 (4.5)	0 (0.0)
Managing on available income, n (%)		
Without problems	12 (54.5)	3 (75.0)
Overall	6 (27.3)	0 (0.0)
Sometimes difficult	4 (18.2)	1 (25.0)
Always difficult	0 (0.0)	0 (0.0)
Declined to answer	0 (0.0)	0 (0.0)
Number of chronic medical diseases		
Mean (SD)	0.82 (1.05)	—^a^
Median (Min-Max)	0.5 (0-3)	—
Level of depressive symptoms (PHQ-9)^b^, n (%)		
Blank	1 (4.5)	—
Mild	1 (4.5)	—
Moderate	17 (77.3)	—
Severe	2 (9.1)	—
Highly severe	1 (4.5)	—
Level of generalized anxiety (GAD-7)^c^, n (%)		
Blank	2 (9.1)	—
Mild	8 (36.4)	—
Moderate	9 (40.9)	—
Severe	3 (13.6)	—

a Not applicable.

b PHQ-9: Patient Health Questionnaire-9.

c GAD-7: Generalized Anxiety Disorder-7.

**Table 2.** Full information maximum likelihood means and SDs.

Variable and role	Mean (SD)
Telepresence T1^a^	
Patient	74.2 (10.99)
Mental health specialist	56.3 (15.73)
Telepresence T2^b^	
Patient	78.2 (14.0)
Mental health specialist	57.7 (17.94)

a T1: time point 1.

b T2: time point 2.

**Table 3.** Actor-partner interdependence model results for actor and partner effects.

Role and effect	Estimate	95% CI	*P* value	Beta (o)	Beta (s)	*r*
Patient						
Intercept	73.90	66.59 to 81.21	<.001	—^a^	—	—
Actor	0.32	−0.19 to 0.82	.21	0.21	0.29	0.25
Partner	−0.06	−0.41 to 0.29	.73	−0.05	−0.07	0.09
k	−0.19	−1.41 to 1.03	—	—	—	—
MHS^b^						
Intercept	70.02	62.0 to 78.04	<.001	—	—	—
Actor	0.93	0.55 to 1.30	<.001	0.78	0.86	0.67
Partner	−0.25	−0.73 to 0.23	.30	−0.21	−0.18	0.31
k	−0.27	−0.82 to 0.28	—	—	—	—

a Not applicable.

b MHS: mental health specialist.

#### Actor Effects

The actor effect for the patients was equal to 0.32 and was not statistically significant (*P*=.21, 95% CI −0.18 to 0.82), and its overall standardized effect was 0.27 (partial *r*=0.25). The actor effect for the MHSs was equal to 0.93 and was statistically significant (*P*<.001, 95% CI 0.55 to 1.3), and the overall standardized actor effect for the MHSs was 0.78 (partial *r*=0.67). When evaluating, if the 2 actor effects were equal, the difference was found not to be statistically significant (*P*=.06; 95% CI −1.23 to 0.02). The overall actor effect was equal to 0.62 and was statistically significant (*P*<.001, 95% CI 0.31 to 0.94).

#### Partner Effects

The partner effect from MHSs to patients was equal to −0.06, which was not statistically significant (*P*=.75, 95% CI −0.41 to 0.29), and its overall standardized effect was −0.05 (partial *r*=−0.09). The partner effect from patients to MHSs was equal to −0.25 and was not statistically significant (*P*=.30, 95% CI −0.73 to 0.22), and its overall standardized partner effect was −0.21 (partial *r*=−0.31). When assessing, if the 2 partner effects were equal, the difference was found not to be statistically significant (*P*=.53, 95% CI −0.4 to 0.78). The overall partner effect was equal to −0.16 and was not statistically significant (*P*=.30, 95% CI −0.45 to 0.14).

#### Dyadic Pattern

We investigated the dyadic pattern by determining the computed ratio of the standardized partner and the actor effect, which needs to be greater than 0.1 in absolute value and statistically significant. The ratio is described by the k parameter. For patients, the value of k was −0.19 (95% CI −1.41 to 1.03), and the k for MHSs was −0.27 (CI −0.82 to 0.28). Due to the nonsignificance of actor and partner effects, we could not find a dyadic pattern for patients. For MHSs, only the actor effect was statistically significant, which suggests an actor-only model (k=0) as the dyadic pattern.

#### Covariates

For patients, the effect of age on telepresence (TVS) at T2 was 0.34 and statistically significant (*P*=.05, 95% CI 0.00 to 0.68), for MHSs the effect was 0.65 and statistically significant (*P*=.04, 95% CI 0.01 to 1.29). The overall standardized effect for age was 0.36.

### Correcting for Unreliability and Sensitivity Analysis

When correcting for unreliability, we did not find any different effect pattern. Moreover, the sensitivity analysis yielded effects comparable to the ones found in the main analysis, which suggests that the actor effect was not conditioned by a single therapist.

## Discussion

### Principal Results

To evaluate the mutual influence within the patient-MHS dyad on the development of telepresence during video consultations, we applied the APIM to dyadic data from a randomized trial assessing the feasibility of MHS video consultations for depression and anxiety disorders in primary care. Our analysis revealed a significant positive actor effect for MHSs, indicating high temporal stability in their experience of telepresence between T1 and T2. In contrast, the actor effect for patients was not statistically significant, suggesting a greater variability in their telepresence between T1 and T2. No significant partner effects were observed, indicating limited mutual influence within dyads.

### Limitations

Several methodological limitations must be acknowledged. First, the fairly small sample size of only 20 dyads, including 4 different MHSs, limits the generalizability of our findings. Despite this, we were able to analyze telepresence data at 2 distinct time points for each dyad, providing a comprehensive understanding of the development of telepresence among patients and MHSs during short intervention video consultations. Second, the patients in our study were randomly allocated to the intervention group, while the control group received treatment as usual from their primary care physicians. This allocation may have led the intervention group patients to perceive themselves as fortunate, potentially enhancing their engagement with the intervention. Consequently, this could have positively influenced their experience of telepresence, leading to an overestimation of its effects. Third, the use of the TVS telepresence scale with professionals has been questioned, particularly regarding the “Absorption” subscale, which may not fully capture the experience of telepresence in task-focused therapists [38].

### Comparison With Prior Work

Regarding the positive actor effect in MHSs, previous studies have shown that perceived telepresence increases with prior experience in video consultations; this effect might be explained by the use of video consultations over several sessions [14,24]. Repeated exposure might strengthen a stable telepresence over time and thus may explain the observed stability in MHS’s telepresence scores (TVS) between T1 and T2.

For patients, the finding of no statistically significant actor effect suggests greater variability or context sensitivity in patients’ telepresence over time. Previous studies have reported high levels of telepresence among patients during video consultations [11,16-18]. The lack of a stable actor’s effect might be explained by depending more on situational factors (eg, content of session, emotional state, technical problems) than on session-invariant factors.

Overall, the average telepresence (TVS) scores were higher in patients compared with MHSs. This may be attributed to the positive impact of receiving specialized treatment within the familiar environment of the primary care setting. Furthermore, values on the absorption subscale were lower for MHSs than for patients, which is in line with the validation study of Cipolletta et al [38].

In our study, we did not find any significant partner effects. One possible explanation is that, with only 4 video consultations conducted, more time may be needed for interdependence in telepresence to emerge. Previous studies have described the strengthening of the working alliance over time in video consultations [41-43]. Additionally, the consultations in our study were limited to diagnostics and a brief intervention, rather than providing comprehensive psychotherapy. This relatively low treatment intensity may have impeded the development of dyadic effects between MHSs and patients.

Another explanation might be that, although telepresence has been identified as a predictor of the therapeutic bond in patients [13], individual-level characteristics such as gender, age, personal skills, technical competence, and environmental conditions rather than dyadic interactions may be more closely linked to the experience of telepresence. For instance, one study found that female gender, professional seniority, employment status, and prior training impacted the feeling of telepresence for MHSs [14]. The effect of age on telepresence is ambiguous; while age has been associated with lower technology literacy and a decreased likelihood of using web-based consultations, it may also correlate with greater professional experience, which could enhance telepresence. However, the reported effect of experience was only small.

### Conclusions

In conclusion, specific factors unrelated to the specific roles in the patient-clinician relationship might impact telepresence for patients and warrant further investigation. Future research should consider larger, more diverse samples and alternative allocation methods to address these limitations and better assess the impact of telepresence in mental health consultations.

Clinically, the high stability of telepresence among MHSs suggests that practitioners may quickly establish a consistent sense of telepresence in video consultations. However, the more variable experience among patients might indicate the importance of individualizing the technical and interpersonal setup of video sessions. Enhancing environmental comfort and technical competence, ensuring privacy, or preparing patients in advance may improve the patient’s sense of telepresence.

## Supplementary material

10.2196/70415 Multimedia Appendix 1 Search strings for the systematic search.

## Abbreviations

APIM: actor-partner interdependence model
MHS: mental health specialist
STROBE: Strengthening the Reporting of Observational Studies in Epidemiology
T1: time point 1
T2: time point 2
TVS: Telepresence in Videoconference Scale
