# Supplementary material for: Development of Telepresence Among Patients and Psychotherapists in the Actor-Partner Interdependence Model: Longitudinal Observational Study of 20 Dyads From a Randomized Trial on Video Consultations in Primary Care
Source: JMIR Ment Health. 2025 Aug 11;12:e70415. doi: 10.2196/70415 (PMC12338751; doi:10.2196/70415)
Supplement: Multimedia Appendix 1 [file mental-v12-e70415-s001.docx]

**APPENDIX 1. SEARCH STRINGS FOR THE SYSTEMATIC SEARCH**

**MEDLINE (through PubMed):**

**Filters:**

Species: Humans; Languages: English, German

**Search string (including filters):**

(telepresen*[Title/Abstract] OR alliance*[Title/Abstract]) AND (telemed*[Title] OR telepsych*[Title] OR video*[Title] OR video-consult*[Title] OR telemental*[Title] OR tele-mental*[Title] OR virtual*[Title])

**Link:**

[*https://pubmed.ncbi.nlm.nih.gov/?term=%28telepresen*%5BTitle%2FAbstract%5D+OR+alliance*%5BTitle%2FAbstract%5D%29+AND+%28telemed*%5BTitle%5D+OR+telepsych*%5BTitle%5D+OR+video*%5BTitle%5D+OR+video-consult*%5BTitle%5D+OR+telemental*%5BTitle%5D+OR+tele-mental*%5BTitle%5D+OR+virtual*%5BTitle%5D%29&filter=hum_ani.humans&filter=lang.english&filter=lang.german&sort=pubdate*](https://pubmed.ncbi.nlm.nih.gov/?term=%28telepresen*%5BTitle%2FAbstract%5D+OR+alliance*%5BTitle%2FAbstract%5D%29+AND+%28telemed*%5BTitle%5D+OR+telepsych*%5BTitle%5D+OR+video*%5BTitle%5D+OR+video-consult*%5BTitle%5D+OR+telemental*%5BTitle%5D+OR+tele-mental*%5BTitle%5D+OR+virtual*%5BTitle%5D%29&filter=hum_ani.humans&filter=lang.english&filter=lang.german&sort=pubdate)

**No. of potentially relevant records:**

227

**WEB OF SCIENCE (Core Collection):**

**Search string:**

(TS=(telepresen* OR alliance*)) AND (TI=(telemed* OR telepsych* OR video* OR video-consult* OR telemental* OR tele-mental* OR virtual*))

**No. of potentially relevant records:**

829
